# *In situ* single-cell therapeutic response imaging facilitated by the TRIPODD fluorescence imaging platform

**DOI:** 10.7150/thno.93256

**Published:** 2024-04-29

**Authors:** Nathan P. McMahon, Allison Solanki, Lei G. Wang, Antonio R. Montaño, Jocelyn A. Jones, Kimberley S. Samkoe, Kenneth M. Tichauer, Summer L. Gibbs

**Affiliations:** 1 Biomedical Engineering Department, Oregon Health & Science University, Portland, OR 97201, USA.; 2 Knight Cancer Institute, Oregon Health & Science University, Portland, OR 97201, USA.; 3 Thayer School of Engineering, Dartmouth College, Hanover, NH 03755, USA.; 4 Geisel School of Medicine, Dartmouth College, Hanover, NH 03755, USA.; 5 Department of Biomedical Engineering, Illinois Institute of Technology, Chicago, IL 60616, USA.

**Keywords:** intracellular paired agent imaging, fluorescence imaging, drug target availability, cyclic immunofluorescence, cancer heterogeneity

## Abstract

**Purpose**: Small molecule drugs such as tyrosine kinase inhibitors (TKIs) targeting tumoral molecular dependencies have become standard of care for numerous cancer types. Notably, epidermal growth factor receptor (EGFR) TKIs (e.g., erlotinib, afatinib, osimertinib) are the current first-line treatment for non-small cell lung cancer (NSCLC) due to their improved therapeutic outcomes for EGFR mutated and overexpressing disease over traditional platinum-based chemotherapy. However, many NSCLC tumors develop resistance to EGFR TKI therapy causing disease progression. Currently, the relationship between *in situ* drug target availability (DTA), local protein expression and therapeutic response cannot be accurately assessed using existing analytical tools despite being crucial to understanding the mechanism of therapeutic efficacy.

**Procedure**: We have previously reported development of our fluorescence imaging platform termed TRIPODD (**T**herapeutic **R**esponse **I**maging through **P**roteomic and **O**ptical **D**rug **D**istribution) that is capable of simultaneous quantification of single-cell DTA and protein expression with preserved spatial context within a tumor. TRIPODD combines two complementary fluorescence imaging techniques: intracellular paired agent imaging (iPAI) to measure DTA and cyclic immunofluorescence (cyCIF), which utilizes oligonucleotide conjugated antibodies (Ab-oligos) for spatial proteomic expression profiling on tissue samples. Herein, TRIPODD was modified and optimized to provide a downstream analysis of therapeutic response through single-cell DTA and proteomic response imaging.

**Results**: We successfully performed sequential imaging of iPAI and cyCIF resulting in high dimensional imaging and biomarker assessment to quantify single-cell DTA and local protein expression on erlotinib treated NSCLC models. Pharmacodynamic and pharmacokinetic studies of the erlotinib iPAI probes revealed that administration of 2.5 mg/kg each of the targeted and untargeted probe 4 h prior to tumor collection enabled calculation of DTA values with high Pearson correlation to EGFR, the erlotinib molecular target, expression in the tumors. Analysis of single-cell biomarker expression revealed that a single erlotinib dose was insufficient to enact a measurable decrease in the EGFR signaling cascade protein expression, where only the DTA metric detected the presence of bound erlotinib.

**Conclusion**: We demonstrated the capability of TRIPODD to evaluate therapeutic response imaging to erlotinib treatment as it relates to signaling inhibition, DTA, proliferation, and apoptosis with preserved spatial context.

## Introduction

The era of targeted cancer therapy has delivered significant progress in improving patient survival rates by selectively targeting genetic and proteomic vulnerabilities present predominantly in malignant tissues. One of the most commonly actionable targets is cell signaling pathway kinases that are deregulated in many cancers 1. Notably, the discovery that ~15% of all non-small cell lung cancers (NSCLC) demonstrate dependance on epidermal growth factor receptor (EGFR) signaling led to the generation of tyrosine kinase inhibitors (TKI) targeting EGFR signaling. Phase III trials consistently showed superior efficacy of first- (e.g., erlotinib) and second-generation (e.g., afatinib) TKIs over standard chemotherapies (e.g., platinum-based) for patients with EGFR-mutated (EGFRmut+) NSCLC, improving progression-free survival (PFS) from 6 months up to 15 months 2-6. However, tumor evolution and subsequent disease progression are still inevitable, most commonly the result of a T790M mutation in EGFR exon 20, which sterically hinders binding of both first- and second-generation EGFR TKIs 7. A third-generation EGFR TKI, osimertinib, overcame this resistance mechanism resulting in superior efficacy and garnered FDA approval in 2018 as a first-line NSCLC treatment option, regardless of T790M mutational status 8, 9. As with other targeted therapies, enthusiasm for initial robust response rates was tempered by inexorable disease progression due to adaptive resistance mechanisms 10. Notably, mechanistic diversity of therapeutic resistance was further reinforced by the 10-20% of EGFRmut+ NSCLC patients that do not respond to first-line EGFR targeted therapy. These *de novo* resistance mechanism(s) are not fully understood, but may relate to *in vivo* pharmacokinetics and could be subverted using combination therapy 11, 12. Additional factors contributing to therapeutic failure include insufficient drug target availability (DTA) at the molecular site of drug-protein interaction and off-target accumulation and activity; however, effective tools to quantify and validate drug delivery and target engagement at the single-cell level are lacking 13, 14. Further, it has been suggested that suboptimal drug dosing regimens may play a role in pushing tumor evolution towards a therapeutically resistant phenotype. However, existing methods for developing drug dosing regimens are not capable of measuring *in situ* DTA to assess how spatial and temporal variation in DTA impact tumor response to therapy 15, 16.

Typically, insufficient DTA and off-target activity are assessed by bulk sampling (e.g., plasma analysis, western blot [WB]), which are not representative of the variable cell-to-cell drug distribution, target binding and off-target effects 17-19. Drug delivery characterization in the context of a highly dynamic tumor microenvironment (e.g., dysfunctional vasculature, hypoxic regions, dense extracellular matrix, immune infiltrate, epithelial to mesenchymal transition, cancer stem cells, etc.), is a key missing component in most drug efficacy studies as it requires *in situ* spatial interpretation that exceeds current analytical capabilities 20. Furthermore, therapeutic efficacy can be correlated to the spatial organization of tumor, immune and stromal cells. As such, significant efforts are underway to interpret therapeutic response within these complex environments 21-25. Numerous small molecule drugs have been labeled with, for example, fluorescent, biotin and photoclickable tags to facilitate direct visualization of drug tissue distribution and target engagement 17, 26-29. Conversely, druggable protein targets have also been genetically modified to enable direct visualization 30, 31. While useful, modifications can vastly alter drug distribution or target engagement, particularly when the label is significantly larger than the drug itself. To overcome this difficulty, various label-free methods have been developed, including mass spectrometry imaging (MSI), cellular thermal shift assay (CETSA) and positron emission tomography (PET) 13, 32-36. However, quantification of available drug targets necessitates accounting for both on-target drug binding and non-specific accumulation in the cells and tissues due to drug affinity, biodistribution, pharmacokinetics, and metabolism 21, 37, 38.

To facilitate quantification of specific and non-specific targeted drug accumulation in tissue, we have adapted a technique from autoradiography termed Paired Agent Imaging (PAI) 39. PAI was created for quantitative *in vivo* imaging, where non-specific accumulation of protein-based, radiolabeled affinity reagents dominated malignant tissue signals but could be corrected for by normalizing the targeted signal to the signal of a co-administered, control antibody labeled with an isotope of different energy 40. In this reinvigorated technique, spectrally-distinct targeted and untargeted imaging probes are used to correct for non-specific uptake to quantify drug target availability (DTA; also termed “binding potential”) 41-57. We have expanded the PAI technique to measure intracellular targets with the use of spectrally-distinct, fluorescently-labeled targeted and untargeted drug derivatives, such as TKIs 58. DTA is calculated by collecting images of targeted and untargeted drug derivatives facilitating calculation of a ratiometric image between the two fluorescent channels. The ratiometric imaging performed in intracellular PAI (iPAI) satisfies the requirement of accounting for both the drug that binds to its target as well as the drug that accumulates in the cells and tissues in a non-specific or untargeted manner enabling spatial DTA calculation. While the goal of a targeted therapeutic is to achieve only on-target binding, in the complex tumor setting there is always some degree of untargeted accumulation that can vary on a cell-by-cell basis in heterogeneous diseases such as NSCLC. Notably, although our technique relies on fluorescently labeled drugs for quantification, all treatment is completed with the parent drug and is thus classified as a label-free method with quantitative assessment of the interaction of the parent drug with its native target.

In addition to assays for directly measuring target engagement, biomarkers are a popular proxy measure for target occupancy that have demonstrated reliable insight into interactions between a drug and its target *in vivo*. If a drug produces an expected therapeutic effect on its target biomarker, then it is assumed that the mechanism was tested and validated as an effective therapeutic. Additionally, common proteomic assays for assessing biomarkers (e.g., conventional immunohistochemistry [IHC] or immunofluorescence [IF]) can provide *in situ* spatial context to therapeutic effect. Historically, these assays were limited to measuring 2-5 antigens per sample due to spectral quantification constraints; however, an evolution of highly multiplexed immunostaining techniques using two distinct methods has emerged: (1) conventional antibody staining (i.e., IF or IHC) in a cyclic fashion 59, 60 or (2) mass spectrometry imaging (MSI) using rare earth metal labeled antibodies 23, 61-71. While both of these approaches provide high-dimensional spatial proteomic tissue maps, they are inherently limited due to their destructive signal removal methods in cyclic imaging workflows and limited sensitivity to low abundance antigens using MSI. In response, we have optimized a technique utilizing oligonucleotide conjugated antibodies (Ab-oligo) to perform cyclic immunofluorescence (cyCIF). In our Ab-oligo cyCIF, a complementary, fluorescently labeled oligonucleotide sequence is used for *in situ* detection 72. We have also demonstrated an Ab-oligo cyCIF signal amplification strategy as well as the flexibility of integrating Ab-oligo cyCIF with both conventional indirect and direct IF reagents for enhanced spatial proteomics assessment 73, 74.

**Figure 1:**
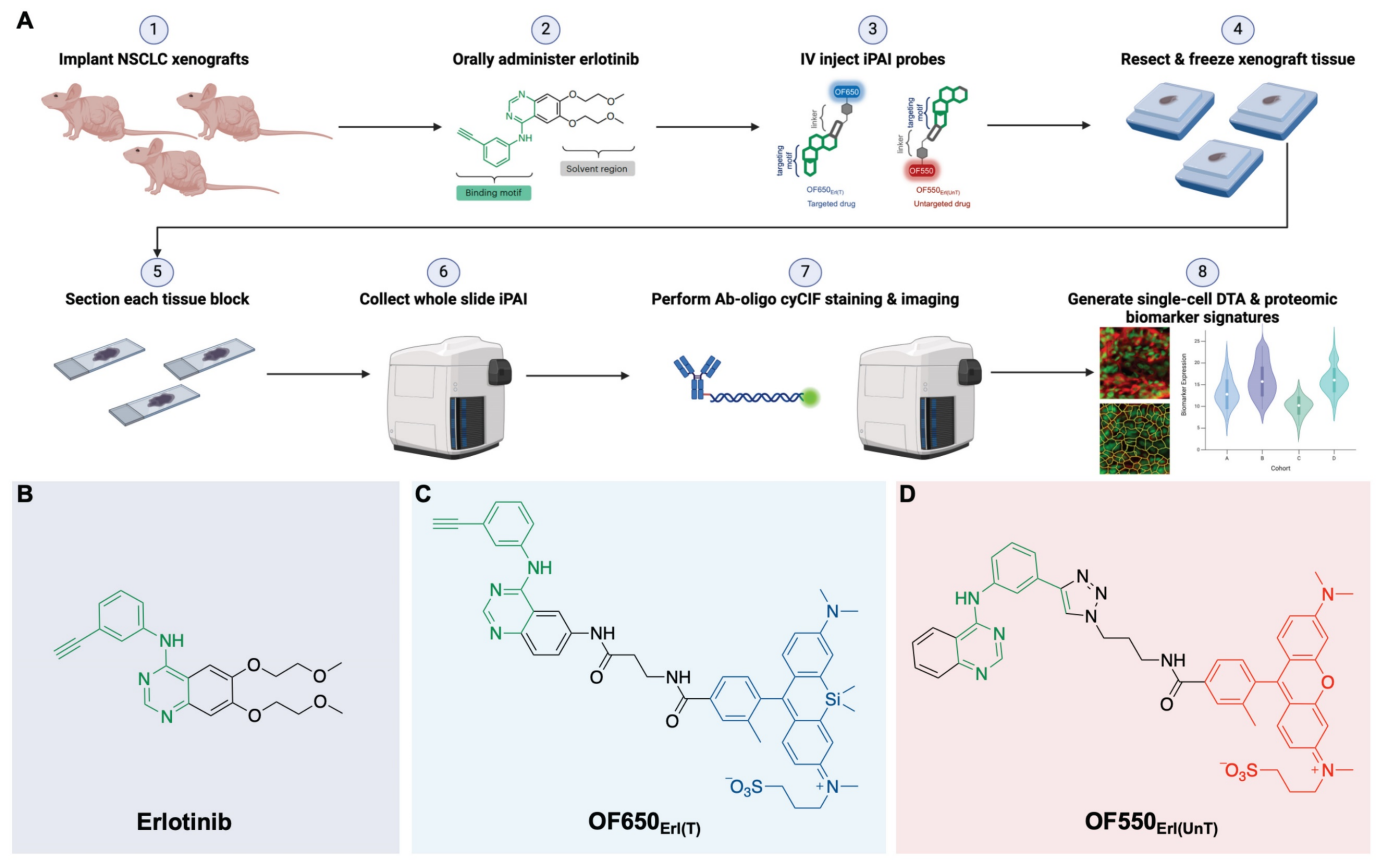
*TRIPODD methodology workflow and administered reagents.* **A.** The TRIPODD workflow is faciliated by (1) growing cell line-derived xenografts (CDX) in athymic nude mice followed by (2) treatment with the parent drug (e.g., erlotinib) and (3) intravenous (IV) administration of the corresponding iPAI probe pair. (4) After tumor resection and preservation as fresh frozen blocks, (5) 10 μm sections were collected for (6) whole slide iPAI microscopy. (7) iPAI probes were removed by saline washes permitting proteomic assessment using Ab-oligo cyCIF and whole slide microscopy of the same tissue section. (8) The iPAI and Ab-oligo cyCIF datasets were combined for downstream analysis of DTA and proteomic biomarker signatures. The small-molecule drug used in the treatment studies and to develop the iPAI probes was **B.** erlotinib, which was fluorescently labeled resulting in the **C.** targeted iPAI probe, OF650_Erl(T)_, and the **D.** untargeted iPAI probe, OF550_Erl(UnT)_.

In summary, the ideal assay to measure therapeutic response requires concomitant measurement of: 1) the extent and heterogeneity of DTA across a tissue as well as 2) drug interactions with the drug target protein along with off-target effects that allow for correlations to be made between efficacy, toxicity and dosing 13. To bridge this unmet analytical gap, we have previously reported our novel fluorescence imaging platform, **T**herapeutic **R**esponse **I**maging through **P**roteomic and **O**ptical **D**rug **D**istribution (**TRIPODD**) 75. TRIPODD facilitates simultaneous single-cell quantification of DTA with iPAI and the associated tumor biology through accurate segmentation of spatially aligned tumor cells based on Ab-oligo cyCIF. To date, TRIPODD is the only methodology to visualize and quantify the complex interactions that define effective cancer therapy. Here we optimized administration of the erlotinib iPAI probes and extended TRIPODD to analyze therapeutic response in EGFRmut+ NSCLC xenografts as measured by changes in DTA and EGFR signaling pathway protein expression demonstrating the capability of TRIPODD to generate a mechanistic understanding of therapeutic response (Fig. 1).

## Materials & Methods

### Overall study design

The overall goals of the studies described herein were to finalize development and validate the quantitative capabilities of the TRIPODD platform on tissues collected from a treatment study. Optimization of the iPAI administration was performed through pharmacodynamic and pharmacokinetic profiling to identify the ideal iPAI probe dose and administration time prior to tissue collection, respectively. Following iPAI administration optimization, a treatment study was performed on NSCLC xenograft bearing mice. The mice were treated with erlotinib (Erl), a first generation, reversible TKI targeting EGFR, followed by iPAI probe administration at various time points within 24 h after Erl treatment. The goal of this study was to assess the relationship between time after Erl administration to therapeutic response as quantified by DTA. A cohort of control mice were also included in these studies that were not treated with Erl. After the completion of the study, xenograft tissues were collected, sectioned and imaged for iPAI probe fluorescence intensity followed by Ab-oligo cyCIF staining and imaging. Downstream analysis was performed on treated and control tissues, permitting the use of the iPAI fluorescence intensities to calculate DTA maps for each tissue sample. The DTA maps were aligned with the Ab-oligo cyCIF images for each tissue sample enabling single-cell quantification of each biomarker using cell segmentation. The single-cell feature data for each biomarker was then visualized to assess the relationship between drug treatment time, DTA and proteomic expression (Fig. 1A). The details of all the methods and reagents are described below.

### Synthesis of fluorescently labeled erlotinib

The targeted and untargeted intracellular paired agent imaging (iPAI) probes used in this study were derivatives of Erl. Briefly, site selection for synthetic modifications of erlotinib to generate the targeted (T) and untargeted (UnT) drug derivatives was guided by the structure of the parent drug bound to the EGFR crystal structure 76, 77. Our novel OregonFluors (OF), OF550 and OF650, were selected for probe labeling due to their spectral separation, overall charge, charge distribution, molecular weight, structural similarity and stability in varied biological environments 58. This new class of tetramethylrhodamine (TMR)/silicon-TMR (Si-TMR) probe derivatives enable maintained biodistribution similarity between the targeted and untargeted probes, while improving stability in varied biological environments. The detailed synthetic procedure for OF550, OF650 and the two iPAI probes labeled with these novel fluorophores, which were utilized in the studies described herein, have been previously published 58. The two iPAI probes were (1) OF650_Erl(T)_ used as the cell membrane permeant iPAI targeted probe with a maximum excitation/emission peak at 649/668 nm and (2) OF550_Erl(UnT)_ used as the cell membrane permeant iPAI untargeted probe with a maximum excitation/emission peak at 552/575 nm (Fig. 1B-D). The targeted and untargeted behavior of the iPAI probes has been extensively validated *in silico*, *in vitro*, and *in vivo* as previously reported 58, 75.

### Antibody conjugation and validation for cyCIF

A 10-antibody panel of oligonucleotide (oligo) and fluorophore conjugated antibodies was developed and staining patterns were validated for the Ab-oligo cyCIF workflow (Table 1). All Ab-oligos, including their oligonucleotide sequences, were generated using the previously reported methods 73, 74. In brief, antibodies to human E-Cadherin (E-Cad), cytokeratin 8 (CK8), EGFR, Akt, pAkt, pMEK, cleaved caspase-3 (CC3) and Ki-67 were purchased from AbCam (Cambridge, UK) and Cell Signaling Technology ([CST], Danvers, MA). A unique dibenzocyclooctyne-terminated (DBCO), single-stranded oligonucleotide (docking strand [DS], 28 mer in length) used to label antibodies, was purchased from Integrated DNA Technologies (IDT, Coralville, IA). Antibody modification and oligonucleotide conjugation were completed with the SiteClick^TM^ Antibody Azido modification kit (ThermoFisher Scientific, Waltham, MA) following the manufacturer's instructions. A primary antibody targeting human MEK1/2 was purchased (AbCam) pre-labeled with Alexa Fluor (AF) 488. A primary antibody reactive to human phospho-EGFR (CST, pEGFR) was purchased and used unconjugated for indirect immunofluorescence with a donkey anti-rabbit secondary (dRb) antibody conjugated to Cyanine-7 (Cy7).

### Cell lines

The human lung adenocarcinoma cell line, HCC827, the human pancreatic ductal adenocarcinoma cell line, PANC-1, and human the colorectal adenocarcinoma cell line, SW620, were purchased from ATCC (Manassas, VA) and maintained mycoplasma free at passage numbers <25 for all studies. The cell lines were expanded in their respective optimal growth media (HCC827: RPMI 1640 [ThermoFisher Scientific] + 10% fetal bovine serum [FBS] + 1% penicillin/streptomycin/glutamine; PANC-1: DMEM [ThermoFisher Scientific] + 10% FBS + 1% penicillin/streptomycin/glutamine; SW620: Leibovitz L-15 [ThermoFisher Scientific] + 10% FBS + 1% penicillin/streptomycin/glutamine) and stored at 37 °C in either a 5% (HCC827 and PANC-1) or 0% (SW620) CO_2_ incubator.

### Animal care and use

All animal experiments were approved by the Oregon Health and Science University (OHSU) Institutional Animal Care and Use Committee (IACUC). All mice were hosted in the AAALAC certified OHSU vivarium, and supplied with food, water and daily inspection to monitor for pain or distress for the duration of experimentation. Mice were placed on a chlorophyll-free diet (Animal Specialties, Inc., Hubbard, OR) one week prior to tumor resection. All rodent surgical procedures, described herein, were performed under full anesthesia composed of a 90/10 mixture of ketamine/xylazine. Ketamine (Hospira Inc., Lake Forest, IL) was administered at a dose of 100 mg/kg and xylazine (AnaSed, Shenandoah, IA) was administered at dose of 10 mg/kg by intraperitoneal (IP) injection. The toe pinch method was employed to verify the depth of anesthesia prior to commencement of any surgical procedures. The standard method of euthanasia for mice was inhalation of carbon dioxide under full anesthesia at the end of each experiment. Euthanasia was confirmed by physical examination to ensure cessation of heartbeat and respiration and is consistent with the recommendations of the Panel on Euthanasia of the American Veterinary Medical Association.

### Mouse xenograft models

Mixed male and female athymic nude mice (Homozygous 490, Charles River Laboratories, Wilmington, MA) were purchased at 32-38 days old. After at least 48 h of acclimation, mice were subcutaneously implanted with HCC827, PANC-1 or SW620 cell xenografts described briefly as follows. Cells were trypsinized, counted and resuspended in their appropriate growth media to a concentration of 2 x 10^7^ cells/ml. The mice were implanted with cells into each rear flank at a final concentration of 1 x 10^6^ cells/flank in 50% v/v Matrigel (Corning Inc., Corning, NY), resulting in two tumors/mouse. A total of n = 35 nude mice were implanted with HCC827 cell line derived xenograft (CDX) models and n = 7 nude mice per cell line were implanted with PANC-1 or SW620 CDX models. The mice were monitored daily after implantation for tumor growth. Tumors were allowed to grow to a maximum size of 1.2 cm^3^ with growth times varying for each cell line (HCC827: ~7-8 weeks; PANC-1 and SW620: ~4 weeks). Mice weighed ~20-25g and tumor volume ranged from 1-1.2 cm^3^ at the time of iPAI probe administration prior to euthanasia and tissue collection.

### Flow cytometry

HCC827, PANC-1 and SW620 cells were trypsinized, counted, and fixed in 4% paraformaldehyde (PFA) for 10 min. A three min permeabilized step (0.5% Triton-X) was followed by 2 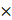 5 min washes in phosphate buffered saline (PBS). 2 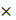
10^6^ cells per cell line were blocked for 15 min with 5% FBS. Without removing blocking buffer, the cells were then incubated with 5 mg/ml cetuximab directly conjugated to AF647 (1:1.7 antibody to fluorophore conjugation ratio). The cells were washed 1 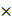 5 min with PBS + 0.1% Tween 20, followed by 2 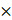 5 min PBS washes, and finally resuspended in fresh PBS prior to analysis on a Becton Dickinson LSR Fortessa (Becton, Dickinson and Company, Franklin Lakes, NJ). The flow cytometer was configured with a 640-1 (670/30) Cy5 channel to detect AF647. A minimum of 1 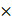 10^5^ cells were counted for each sample. To quantify EGFR receptor number, Quantum^TM^ Alexa Fluor® 647 molecules of equivalent soluble fluorophore (MESF) beads (Bangs Laboratories, Inc., Fishers, IN) were quantified prior to the cellular samples.

### Fluorescence microscopy of tissue sections

All fluorescence microscopy of tissue sections was performed using the following methods. Frozen tissue blocks were cryosectioned at 10 μm thickness (Leica Biosystems, Wetzlar, Germany) onto SuperFrost Plus glass slides (ThermoFisher). Fluorescence images of whole tissue sections were acquired on a Zeiss AxioScan.Z1 microscope (Carl Zeiss AG, Oberkochen, Germany) equipped with a Colibri 7 light source (Carl Zeiss AG) and Orca Flash4 v.2 camera (Hamamatsu Photonics, Hamamatsu, Shizouka, Japan). Images were collected using the following filter sets: Zeiss 38HE (Cy2/AF488 [Carl Zeiss AG]), Zeiss 43HE (Cy3/AF555 [Carl Zeiss AG]), Zeiss 50 (Cy5/AF647 [Carl Zeiss AG]) and Chroma 49007-ET-Cy7 (Cy7/AF750 [Chroma Technology Corporation, Bellows Falls, VT]). Excitation light was filtered using the following bandpass (BP) filters: 470/40 (38HE), 550/25 (43HE), 640/30 (50) and 710/75 (49007-ET-Cy7) for Cy2, Cy3, Cy5 and Cy7 channels, respectively. Emission light was filtered using the following BP filters: 525/50 (38HE), 605/70 (43HE), 690/50 (50) and 810/90 (49007-ET-Cy7) for Cy2, Cy3, Cy5 and Cy7 channels, respectively. DAPI was imaged using a Semrock filter set (Zeiss), where excitation was filtered at 405 nm and emission was filtered using a 410/480 BP filter. Images were captured at 20X (Plan-Apochromat, 0.8NA) magnification, where image tiles with 10% overlap were stitched together using the Zeiss Zen software to produce a single tissue map.

### Pharmacokinetic study of iPAI probes

To characterize the pharmacokinetic activity of iPAI probes, the OF650_Erl(T)_ and OF550_Erl(UnT)_ iPAI probes were injected simultaneously via tail vein injection to n = 18 HCC827 CDX bearing mice at 2.5 mg/kg for both the targeted and untargeted iPAI probes. Mice (n = 3 per timepoint) were sacrificed and tumors resected and flash frozen in optimal cutting temperature (OCT) compound at 15, 30, 60, 120, 240 or 480 min after iPAI probe injection. Injection vehicle for all systemically administered TKI and iPAI probes was a co-solvent mixture of a ratio of 10% dimethyl sulfoxide ([DMSO], Sigma-Aldrich, St. Louis, MO), 5% Kolliphor (Sigma-Aldrich), and 85% of 75% FBS/PBS (VWR Scientific). A 10 µm thick tissue section from each frozen tissue block was collected and imaged as described above. The targeted iPAI probe was imaged in the Cy5 channel, the untargeted iPAI probe was imaged in the Cy3 channel and tissue autofluorescence imaged in the Cy2 channel was used to generate a focus map. After image acquisition, a custom MATLAB script (10.5281/zenodo.4004647) was used to calculate ratiometic DTA images on a per pixel basis for each tissue image. DTA, as previously reported 75, was calculated as


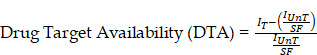

(1)

where I_T_ is the targeted iPAI probe fluorescence intensity, I_UnT_ is the untargeted iPAI probe fluorescence intensity and SF is a scaling factor to account for fluorescence signal intensity difference between the target and untargeted probes. The scaling factor was quantified by imaging the targeted and untargeted probes at titrated concentrations on the Zeiss AxioScan.Z1 to generate a linear calibration trend line for each probe, where the scaling factor was calculated as the ratio of the slope of the untargeted probe trend line to the slope of the targeted probe trend line and was built into the MATLAB analysis script. Targeted probe, untargeted probe and DTA signal for each tissue section was quantified by manual segmentation of the tissue in ImageJ v1.51 (National Institute of Health, Bethesda, MD) and the mean value for each channel from the tissue sections was extracted. The mean fluorescence signal or DTA value from each tissue section was divided by the highest mean fluorescence or DTA value, respectively, to calculate relative fluorescence intensity or DTA values. Box and whisker plots comparing tissue sections resected at different times after iPAI probe injection were generated in Prism v9.0 (GraphPad, San Diego, CA).

### Pharmacodynamic study of iPAI probes

The pharmacodynamic profiles of iPAI probes were assessed in HCC827, PANC-1 and SW620 CDX mouse models, where the targeted and untargeted iPAI probes were simultaneously administered via tail vein injections at 5, 2.5 or 1.25 mg/kg for both targeted and untargeted iPAI probes. For equivalent group size comparison, each dose group was comprised of n = 2 mice per CDX model. Injection vehicle for all systemically administered TKI and iPAI probes was a co-solvent mixture of 10% DMSO, 5% Kolliphor, and 85% of 75% FBS/PBS. For all CDX models, tumors were resected 4 h after systemic administration and flash frozen in OCT compound. An additional n = 1 mouse per CDX model was injected with co-solvent vehicle only to quantify tissue autofluorescence in each CDX model. A 10 µm thick tissue section was collected from each frozen tissue block and imaged on a Zeiss AxioScan.Z1, where images were collected in the Cy5 (targeted iPAI probe), Cy3 (untargeted iPAI probe) and Cy2 (autofluorescence used to generate focus map) channels. Mean fluorescence intensity for each image was calculated in ImageJ through manual segmentation of the tissue image. The relative fluorescence intensities for each image were calculated by dividing the mean fluorescence intensity of an image by the highest mean fluorescence intensity of that particular image channel (i.e., Cy5 and Cy3). The previously described MATLAB script was used to calculate DTA images for the HCC827, PANC-1 and SW620 iPAI injected tissues (Equation 1). Box and whisker plots comparing tumor types were generated in Prism v9.0.

To identify the iPAI dose that provided quantitative imaging, the targeted probe, untargeted probe and DTA signals on all tissue sections collected above were correlated to their EGFR and pEGFR protein expression as measured by indirect immunofluorescence. Following iPAI microscopy of tissue sections of all tumors from all three CDX models in all dose groups (n=2 mice/CDX/dose group [6 mice/CDX]), iPAI probes were removed with 3 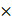 5 min saline washes to render the tissue available for immunofluorescent staining. Each tissue section was then stained following the protocol described below with primary EGFR (Cetuximab, Eli Lilly, Indianapolis, IN) and pEGFR antibodies. The stained slides were mounted in Fluoromount-G (Southern Biotech, Birmingham, AL) and cover-slipped for imaging. The stained slides were imaged on a Zeiss AxioScan.Z1. Images were collected in the DAPI, Cy3 and Cy5 channels. EGFR and pEGFR signals for each tissue section were quantified by manual segmentation of the tissue in ImageJ v1.51, where the mean value for each channel was calculated. Scatter plots and Pearson's correlations for all dose groups correlating EGFR and pEGFR expression to targeted probe, untargeted probe and DTA signal were generated in Prism v9.0.

### Erlotinib treatment of HCC827 xenografts

TRIPODD was used to investigate the relationship between erlotinib dosing regimen and therapeutic response to erlotinib in HCC827 CDX bearing mice. Erlotinib was dissolved in 0.3% weight/volume of sodium carboxymethylcellulose (Sigma-Aldrich) and 0.1% volume/volume of Tween 80 (Sigma-Aldrich) in PBS (VWR Scientific) and administered by oral gavage as a single dose of 50 mg/kg when HCC827 CDX tumors reached a minimum size of 150 mm^3^ 78, 79. Control and treatment cohorts were created so that n = 12 HCC827 CDX mice were randomly assigned to a treatment group (n = 9 mice) or the untreated, control group (n = 3 mice). The n = 9 mice selected for treatment were further randomly divided into groups where mice were euthanized at 6, 12 or 24 h (n = 3 per timepoint) after erlotinib administration followed by tumor resection. iPAI reagents, OF650_Erl(T)_ and OF550_Erl(UnT)_, were co-administered systemically via tail vein injection at a dose of 2.5 mg/kg for each probe 4 h prior to euthanasia. After resection, all tumors (n = 36 tumors, 2 tumors/mouse) were flash frozen in OCT compound.

### DTA quantification of erlotinib treatment study

A 10 µm thick tissue section was collected from each frozen tissue block and imaged on a Zeiss AxioScan.Z1, where images were collected in the Cy5 (targeted iPAI probe), Cy3 (untargeted iPAI probe) and Cy2 (autofluorescence used to generate focus map) channels. The previously described MATLAB script was used to calculate ratiometric DTA images for tissue sections (Equation 1) 75.

### Ab-oligo cyCIF imaging of erlotinib treatment kinetics

Following iPAI microscopy of tissue sections, iPAI probes were removed with 3 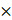 5 min saline wash to render the tissue available for Ab-oligo cyCIF imaging. Each tissue section was incubated in 2% PFA at room temperature (RT) for 15 min and then washed with 1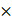 PBS, pH 7.4 (3 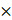 5 min). The tissue was then permeabilized using 1X PBS, pH 7.4 + 0.3% Triton X-100 for 15 min at RT and washed with pH 7.4 PBS (3 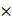 5 min). The slides were blocked at RT for 30 min in Ab-oligo blocking and dilution buffer which contained 2% bovine serum albumin (BSA, bioWORLD, Dublin, OH), 0.5 mg/mL sheared salmon sperm DNA (ThermoFisher) and 0.5% dextran sulfate (Sigma-Aldrich) in 1X PBS, pH 7.4. Each tissue section was covered with 40 μL of unconjugated pEGFR antibody diluted to a concentration of 15 μg/mL and incubated for 1 h at RT. An additional tissue section was incubated with blocking buffer without primary antibody to serve as a negative control. Excess pEGFR antibody was removed by washing with 2X SSC buffer, pH 7 for 3 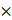 5 min. Each tissue section was then fixed using a 15 min incubation of 2% PFA at RT and washed with 2X SSC pH 7 (3 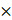 5 min). pEGFR was labeled with dRb secondary antibody conjugated to Cy7. The secondary antibodies were diluted in dilution buffer containing 2% BSA, 0.5 mg/mL sheared salmon sperm DNA and 0.5% dextran sulfate in 2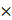 SSC buffer. The dRb-Cy7 was diluted to a final protein concentration of 350 nM. Each tissue section, including the negative control, was covered with 40 μL of the diluted secondary antibody and incubated for 45 min at RT in a humidified chamber protected from light. Excess secondary antibody was removed by washing with 2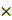 SSC buffer for 3 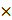 5 min. Then to facilitate Ab-oligo cyCIF imaging, the eight Ab-oligo conjugates (Table 1) were mixed at a concentration of 15 μg/mL per antibody into a single cocktail. Each tissue section was covered with 40 μL of the diluted antibody cocktail and incubated at 4 °C overnight in a humidified chamber. The negative control slide was again incubated with blocking buffer without any antibody. The next day, the sections were washed with 2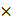 SSC buffer for 3 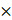 5 min. The sections were fixed in 2% PFA for 15 min at RT, then washed again in 2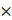 SSC buffer (3 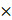 5 min).

Cycles of imaging strand (IS) application, image collection and signal removal were performed to specifically label E-Cad, Ki67, pAkt, CK8, CC3, pMEK, EGFR, and Akt. Each marker was fluorescently labeled using one of two unique IS labeling strategies. The first strategy employed the use of a 26 nucleotide (nt) IS with a fluorophore conjugated through a photocleavable linker (PCL) at both the 3' and 5' ends of the oligo (2xPCL-FL IS) 72. This strategy was applied for labeling E-Cad, Ki67, CK8 and EGFR. A second strategy capable of amplifying the fluorescence was incorporated into this study to label pAkt, CC3, pMEK and Akt 74. Importantly, these two strategies were used simultaneously in rounds of imaging as follows. First, the amplification strand (AmpS) for all markers to be imaged in a given round were diluted to 350 nM in dilution buffer containing 2% BSA, 0.5 mg/mL sheared salmon sperm DNA and 0.5% dextran sulfate in 2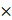 SSC buffer. The mixture was then heated to 85 °C for 3 min to decrease formation of any secondary structures. Then any 26 nt, 2xPCL-FL IS were added to the mixture for a final concentration of 350 nM of each IS. 40 μL of the diluted oligo cocktail was applied to each slide and incubated at RT for 45 min while protected from light. Unbound 26 nt IS and AmpS was removed by washing with 2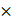 SSC buffer (3 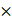 5 min). Then the amplification IS (Amp IS) was diluted to a final concentration of 7 μM and 40 μL dispensed onto each tissue. The Amp IS was incubated at RT for 45 min while protected from light. Unbound Amp IS was removed by washing with 2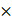 SSC buffer (3 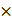 5 min). DAPI was then applied to all stained samples at 300 nM for 10 min at RT and the samples were washed in 2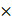 SSC buffer (2 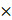 5 min). All stained slides were mounted with Fluoromount-G and cover-slipped for imaging.

Images were collected on a Zeiss AxioScan.Z1 in the DAPI, Cy2, Cy3, Cy5, and Cy7 channels for each round of staining. All stained and imaged slides were treated with UV light for 15 min followed by washing 10 times with 0.1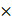 SSC and remounted with Fluoromount-G. Finally, the slides were imaged with the same settings used prior to UV treatment to confirm complete signal removal. Subsequent rounds of IS addition, imaging and signal removal were repeated until all Ab-oligo conjugates were imaged.

Autofluorescence images for background subtraction were acquired after collection of all antibody marker imaging data by removing coverslips and quenching the fluorescence by incubating the slides for 30 min in a solution of 3% peroxide and 20 mM sodium hydroxide (NaOH) in PBS. The slides were then labeled with DAPI, mounted in Fluoromount-G and cover-slipped for imaging. The slides were imaged again on a Zeiss AxioScan.Z1 in the DAPI, Cy2, Cy3, Cy5 and Cy7 channels to collect autofluorescence images for background subtraction in downstream analysis.

### TRIPODD analysis of erlotinib therapeutic response kinetics

The iPAI and antibody imaging datasets for each tissue section were spatially registered. Images acquired during cyCIF were registered based on DAPI features imaged in each round of staining using a custom MATLAB script 80. The generated DTA tissue map for each tissue section was manually registered to the EGFR image, due to pattern similarity of DTA images, with the built-in ImageJ registration plugin, “Align image by line ROI”. Cell segmentation, feature extraction and image visualization of the fully registered dataset was performed with QiTissue Software (Quantitative Imaging Systems, LLC, Pittsburg, PA). A previously reported analysis pipeline was then utilized for feature extraction and data filtering 81. Briefly, the features that were extracted included nuclear size and mean intensity for each marker, including autofluorescence in each wavelength. Autofluorescence in the Cy3 channel and nuclear size were used to filter outliers cells, where cells >95^th^ quantile for Cy3 autofluorescence were removed and cells with nuclear sizes <5^th^ quantile and >95^th^ quantile were removed. These removed cells were flagged as outliers since they possessed either high autofluorescence suggesting tissue artifact or their size was unlikely to be an accurately segmented nucleus in a xenograft tissue sample comprised of largely the same cell type, which are of similar size. Additionally, only xenograft cancer cells were selected for downstream analysis by filtering for cells expressing the epithelial markers, CK8 and E-Cad.

Autofluorescence background subtraction was performed by dividing all autofluorescence and marker mean intensities by their respective exposure time. The autofluorescence value was subtracted from each cell's biomarker value based on the respective wavelength in which the marker was imaged. To scale all fluorescence intensity for cyCIF and DTA calculations, the data for each biomarker was z-scored using the median and standard deviation across all cells from the untreated control tissues for each biomarker. Violin plots were generated to evaluate changes in biomarker expression based on treatment cohort using a custom python script.

### Statistical analysis

To facilitate pharmacokinetic evaluation of iPAI probes, fluorescence intensity of iPAI and calculated DTA from HCC827 xenografts collected at each timepoint were compared to HCC827 xenografts administered only injection vehicle. To enable pharmacodynamic evaluation of iPAI probes, comparison of the iPAI fluorescence intensity and calculated DTA at each iPAI probe dose in xenograft tissues with positive EGFR expression, PANC-1 and HCC827, were compared to all other iPAI doses as well as xenograft tissue where only injection vehicle was administered. In all analyses, significance was evaluated using a one-way analysis of variance (ANOVA) followed by Fisher's least significant difference (LSD) multiple comparison test. The α value was set to 0.05 for all analyses. P-values for significance are denoted by the number of asterisks above the box and whisker plot as follows: *p < 0.05, **p < 0.01, ***p < 0.001, and ****p < 0.0001. All statistical analyses were completed using Prism v9.0.

## Results

### Pharmacokinetic evaluation of iPAI probes in HCC827 xenografts

**Figure 2:**
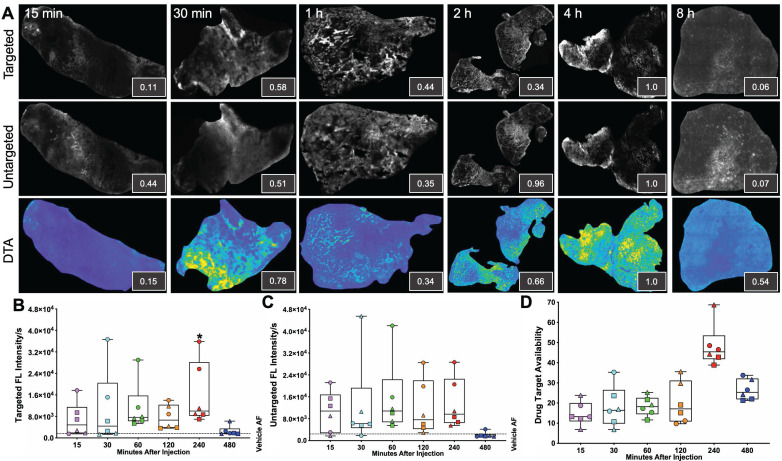
*Pharmacokinetic characterization of iPAI probe tissue fluorescence intensity and DTA.* HCC827 CDX tumors were resected 15, 30, 60, 120, 240 or 480 min after the mice were systemically administered 2.5 mg/kg each of the targeted and untargeted iPAI probes. **A.** Representative fluorescence images of tumor tissue sections (n=4 tumors per timepoint) of the targeted, untargeted and calculated DTA tissue maps. To enable fluorescence signal spatial pattern visualization, all the targeted and untargeted images are displayed with auto-contrast levels for visualization while the DTA tissue maps are scaled equivalently. Relative signal intensities for each imaging channel (insets) for each representative tissue section were calculated. Assessment of **B.** targeted and **C.** untargeted probe uptake as well as **D.** DTA variability across all analyzed tumors was performed. Points of the same shape signify that the tumors were collected from the same mouse at each timepoint. The black dashed line indicates the level of tissue autofluorescence as quantified from HCC827 xenograft tissue following a vehicle only injection. The timepoints were compared to vehicle autofluorescence (AF) using a one-way ANOVA, where significance is denoted by the number of asterisks as follows: *p < 0.05.

iPAI probe pharmacokinetics (PK) were characterized to facilitate resection of tumor tissues with the highest accumulated fluorescence intensity to permit the greatest dynamic range for DTA map calculations. The iPAI probe PK profiles were evaluated in HCC827 xenograft tissues that were resected 15, 30, 60, 120, 240, or 480 min after systemic administration of 2.5 mg/kg each of the targeted and untargeted iPAI probes (Fig. 2). Whole tissue section fluorescence images from tumors (n = 3 mice/timepoint, 2 tumors/mouse) were used to quantify the mean signal intensities in the targeted (Cy5 - OF650_Erl[T]_) and untargeted (Cy3 - OF550_Erl[UnT]_) channels, facilitating calculation of the DTA tissue maps and mean DTA for each image. Variation in the spatial localization of the targeted and untargeted probe distribution was observed across evaluated timepoints. Notably, the fluorescence intensity of the targeted probe was highest at the 240 min timepoint. The untargeted fluorescence intensity was also highest at the 240 min timepoint, which was not substantially different than the untargeted fluorescence intensity at the 120-min timepoint. The calculated DTA was substantially higher at the 240 min timepoint as compared to all other evaluated timepoints (Fig. 2A). Quantification across tissue sections from each tumor within the timepoint group showed similar results where, the highest median targeted fluorescence was at the 240 min timepoint, which was also significantly greater than autofluorescence intensity in the vehicle injected control group (Fig. 2B). Similar median intensities were seen in the untargeted channel at 60, 120 and 240 min with no significant difference observed between any timepoint and vehicle autofluorescence. Notably, for the untargeted fluorescence intensity, one tissue section sampled at the 120 min timepoint had fluorescence equivalent to tissue autofluorescence level, showing high variance in the untargeted probe accumulation (Fig. 2C). The calculated DTA maps, demonstrated that the 240 min timepoint resulted in the highest median DTA from all tissue sections (Fig. 2D) and was thus selected as the optimal PK timepoint for future studies.

### Pharmacodynamic assessment of iPAI probes in varied EGFR expressing tissues

**Figure 3:**
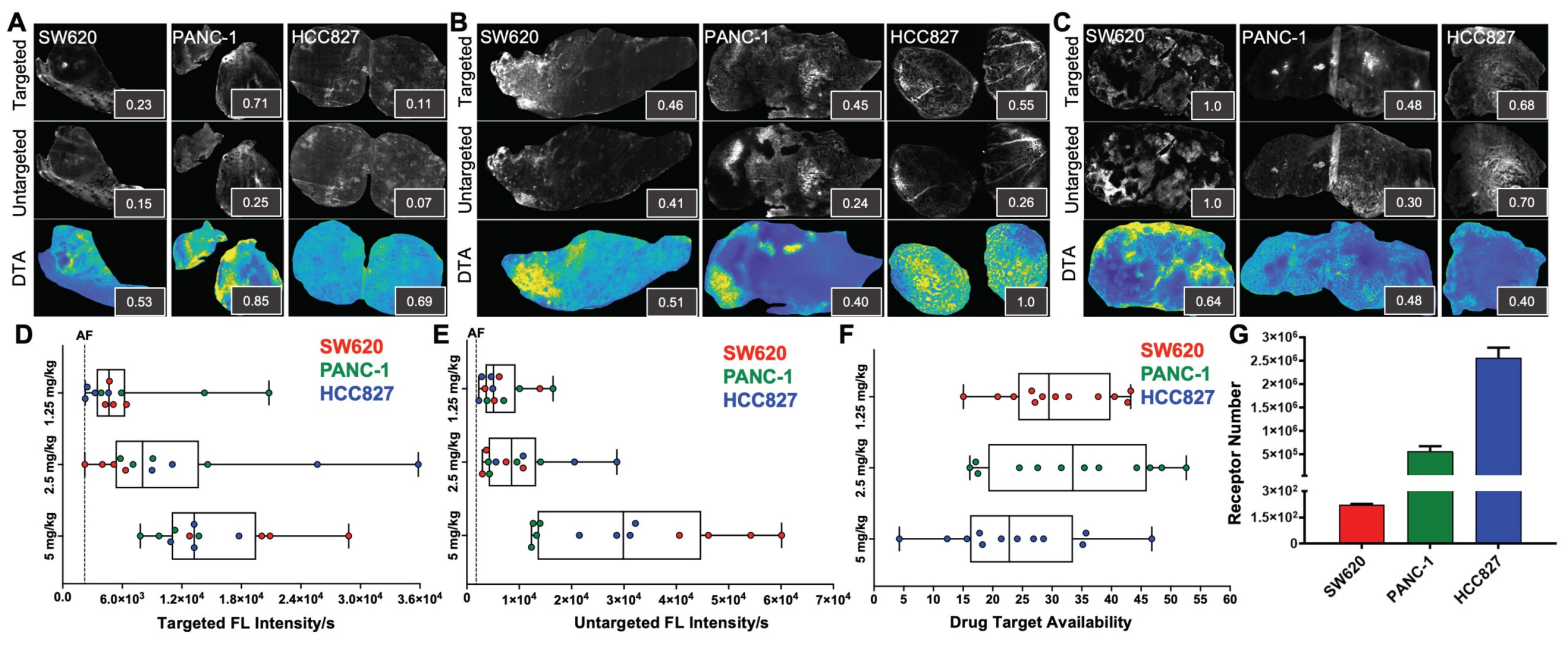
*Pharmacodynamic assessment of iPAI probes in xenografts with varied EGFR expression*. Mice bearing HCC827, PANC-1 and SW620 CDX tumors were systemically administered **A.** 1.25 mg/kg, **B.** 2.5 mg/kg or **C.** 5 mg/kg of the targeted and untargeted iPAI probes, which were resected 4 h after injection. Representative images of targeted and untargeted probe uptake from (n=4 tumors/CDX/dose) HCC827, PANC-1 and SW620 tissue sections displayed with auto-contrast, where inset values represent relative signal intensity normalized across imaging channels. Targeted and untargeted tissue images enabled calculation of DTA spatial maps and relative DTA values (insets) for each representative tissue section with all images displayed at the same contrast levels. Assessment of **D.** targeted and **E.** untargeted probe uptake as well as **F.** DTA variability across all analyzed tumors was completed. The black dashed line indicates the average level of tissue autofluorescence (AF) as quantified from HCC827, PANC-1 and SW620 xenograft tissues (n=2 tumors per cell line) following a vehicle only injection. **G.** Quantification of EGFR expression in SW620, PANC-1 and HCC827 cells was performed by flow cytometry.

iPAI probe pharmacodynamic (PD) activity was assessed to optimize iPAI probe dose and permit the greatest dynamic range for DTA tissue map calculations. The iPAI PD activity was quantified in xenograft tissues with varied endogenous EGFR expression *in vitro* to assess the effect of varied iPAI target protein levels on DTA calculations (Fig. 3). EGFR number for each cell line, as quantified by flow cytometry, demonstrated high EGFR expression for the HCC827 (average EGFR per cell = 2.4 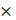
10^6^), mid-level EGFR expression for the PANC-1 (average EGFR per cell = 6.7 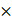 10^5^) and low EGFR expression for the SW620 (average EGFR per cell = 2.3 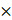 10^2^) cell lines (Fig. 3G). Whole tissue section fluorescence images were collected from each distinct CDX cohort grown from the cell lines with varied EGFR expression after administration of 1.25, 2.5 or 5 mg/kg of each iPAI probe (n = 4 mice per dose, 2 tumors/mouse). The mean fluorescence intensities for the targeted (Cy5 - OF650_Erl[T]_) and untargeted (Cy3 - OF550_Erl[UnT]_) probes were qualitatively and quantitatively evaluated to select an optimal iPAI probe dose. The targeted and untargeted fluorescence images were used to both generate DTA maps and quantify mean DTA for each tumor. Relative fluorescence and DTA values were calculated per imaging channel per CDX type (Fig. 3A-C). In the 1.25-mg/kg dose group, PANC-1 xenografts had the highest relative targeted and untargeted iPAI probe fluorescence intensity as well as the highest relative DTA (Fig. 3A). The representative image of the HCC827 xenograft tumor showed the highest relative targeted fluorescence in the 2.5 mg/kg cohort as well as the highest relative DTA of all displayed tissue sections (Fig. 3B). The displayed SW620 xenograft in the 2.5 mg/kg dose group resulted in the highest relative untargeted fluorescence within the dose group. The 5 mg/kg dose group showed the highest relative targeted and untargeted fluorescence intensities from the displayed SW620 xenograft section (Fig. 3C). The SW620 xenograft sections also resulted in the highest relative DTA in comparison to the displayed PANC-1 and HCC827 xenograft sections in the 5 mg/kg dose group. The lower quartile, median, and upper quartile values were calculated from the targeted, untargeted and DTA images for all xenograft tissues in all dose groups (Fig. 3D-F). For the targeted and untargeted probes, a positively correlated dose dependent relationship with the median was found (Fig. 3D & 3E). For the calculated DTA, the highest median and maximum DTA values were found in the 2.5 mg/kg dose group (Fig. 3F).

**Figure 4:**
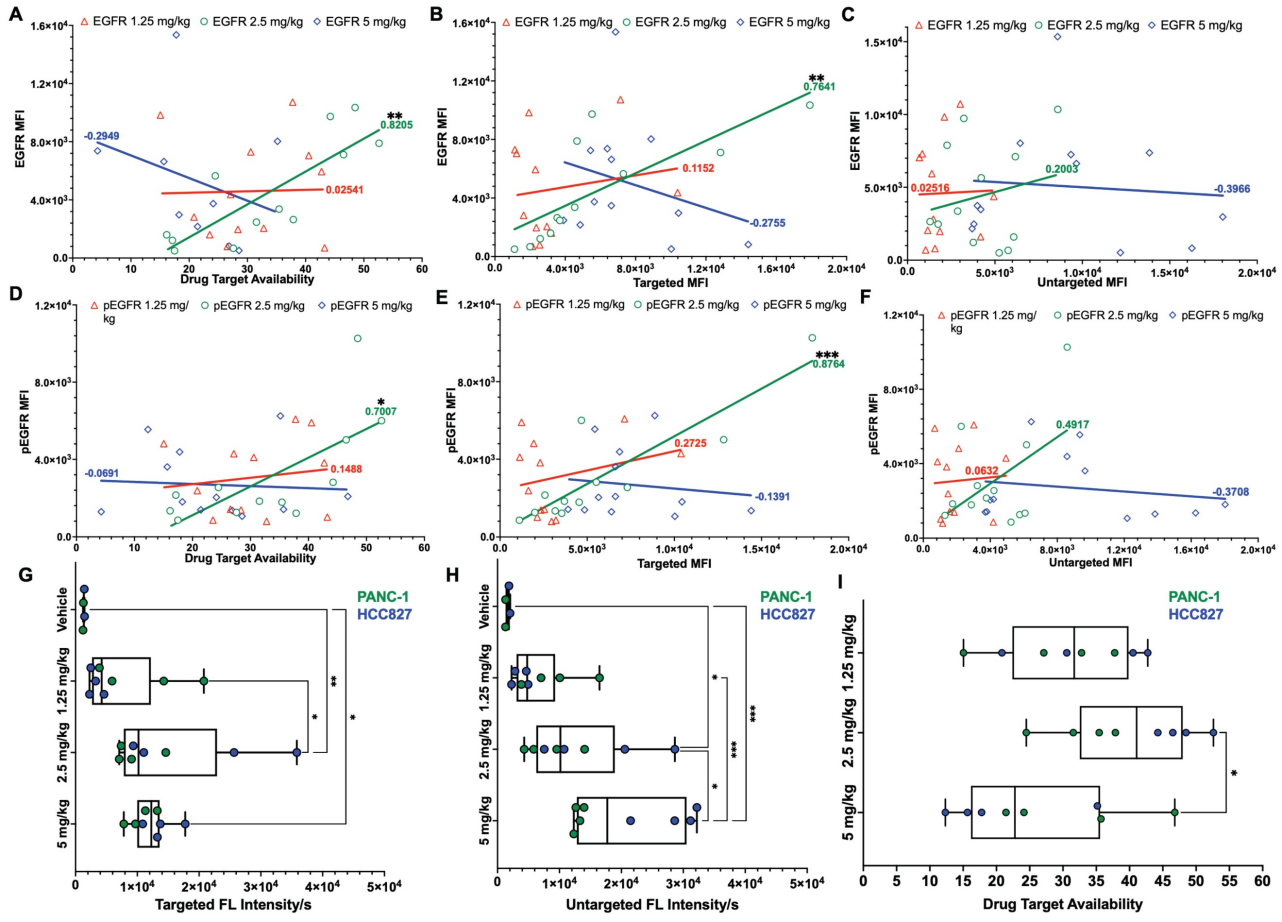
Pearson's correlation of iPAI signal to EGFR expression and pharmacodynamic assessment of iPAI probes in EGFR-positive xenografts. Pearson correlation coefficients were calculated to compare EGFR expression to **A.** DTA, **B.** targeted probe and **C.** untargeted probe mean fluorescence intensity (MFI). Pearson correlation coefficients were also calculated to study the relationship between pEGFR expression and **D.** DTA, **E.** targeted probe and **F.** untargeted probe MFI. Solid lines represent the linear regression trendline and are the color of their respective dose group. Calculated correlation coefficients per dose group are also color coded using the same scheme. HCC827 and PANC-1 CDX bearing mice were injected with either vehicle, 1.25 mg/kg, 2.5 mg/kg, or 5 mg/kg of iPAI probes and **G.** targeted probe fluorescence intensity, **H.** untargeted fluorescence intensity and **I.** DTA were measured. Significance in all plots is denoted by the number of asterisks as follows: *p < 0.05, **p < 0.01 and ***p < 0.001.

To assess the relationship between iPAI dose and EGFR expression, all tissues from all CDXs were immunostained to quantify EGFR and pEGFR expression. Pearson's correlation coefficients were calculated and scatterplots were utilized to compare EGFR and pEGFR expression to DTA, targeted probe and untargeted probe fluorescence signals (Fig. 4). In the DTA and targeted imaging channels, only the 2.5 mg/kg dose group demonstrated significant correlation between EGFR and pEGFR expression to either DTA or targeted probe signal (Fig. 4A, 4B, 4D & 4E). In the untargeted probe imaging channel, there were no significant correlations calculated between the untargeted probe signal to EGFR or pEGFR expression in any dose group (Fig. 4C & 4F). Comparison of iPAI probe intensity to injection vehicle autofluorescence was also performed with EGFR-positive xenograft tissues, HCC827 and PANC-1. To avoid data artifact caused by EGFR-negative tissues, where there were minimal targeted iPAI probe binding sites, the SW620 xenograft tissues were excluded from this analysis. The targeted probe fluorescence intensity was significantly greater than vehicle-injected xenografts in the 2.5 mg/kg and 5 mg/kg dose cohorts, while the 1.25 mg/kg dose cohort was not significantly different than vehicle injected control autofluorescence (Fig. 4G). Additionally, there was no statistical difference in targeted probe fluorescence intensity between the 2.5 mg/kg and 5 mg/kg cohorts. The same trend was seen in the untargeted probe imaging channel, where the 1.25 mg/kg cohort was not significantly greater than vehicle injected control autofluorescence, while the 2.5 mg/kg and 5 mg/kg cohorts were significantly greater than vehicle injected control autofluorescence (Fig. 4H). The median DTA was observed to be greatest in the 2.5 mg/kg cohort, which was only significantly greater than the 5 mg/kg cohort median DTA (Fig. 4I). Thus, the 2.5 mg/kg dose was selected as the optimal iPAI probe dose for future studies.

### TRIPODD analysis of single- and multi-dosed HCC827 xenografts

**Figure 5:**
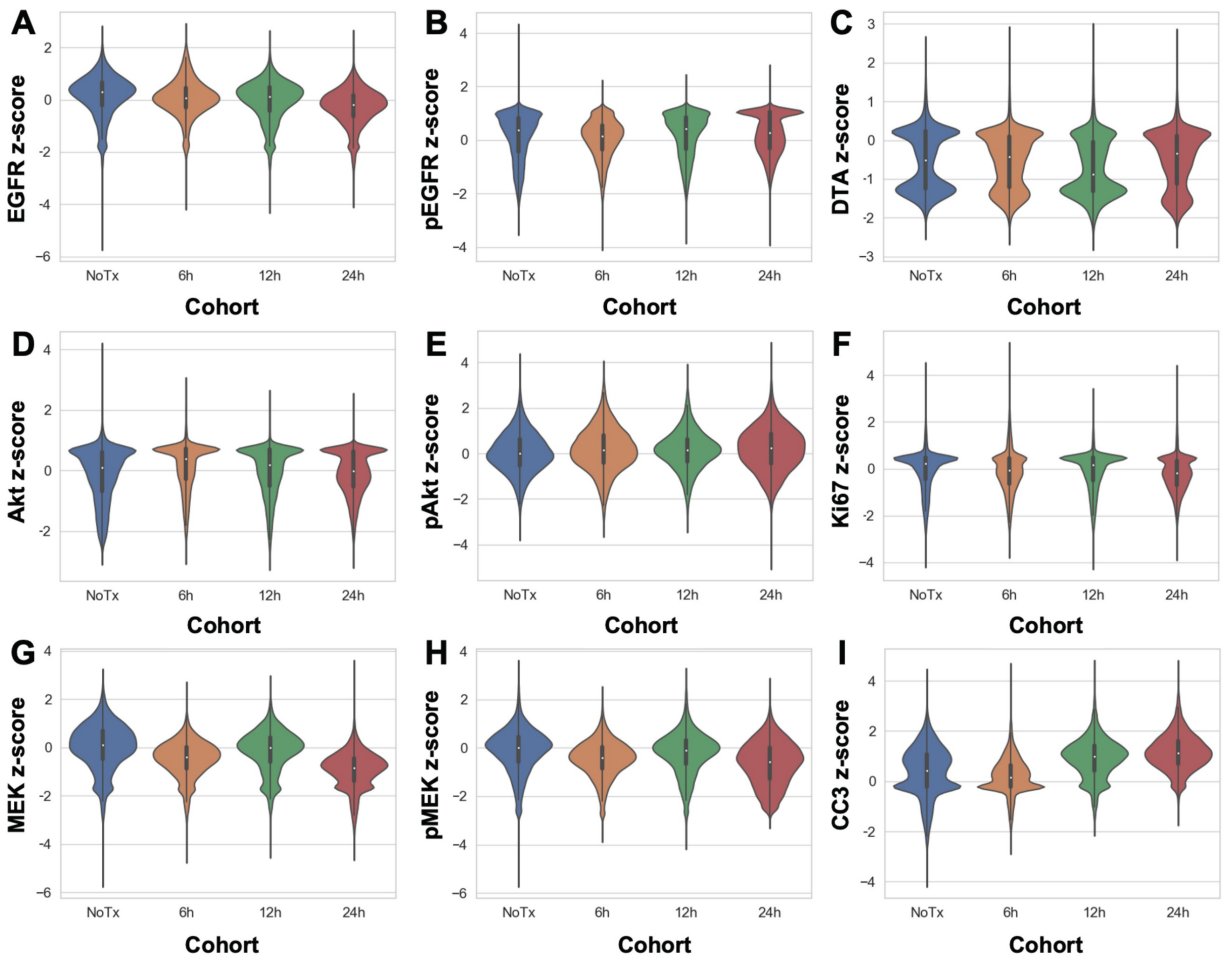
*Single-cell quantification of erlotinib therapeutic response and DTA.* The single-cell z-scored expressions for each biomarker in each treatment cohort were combined to compare the control, untreated cohort (NoTx) to the erlotinib therapeutic response measured in the erlotinib treated cohorts (i.e., 6h, 12h or 24h). The signal levels of **A.** EGFR, **B.** pEGFR, **C.** DTA, **D.** Akt, **E.** pAkt, **F.** Ki67, **G.** MEK, **H.** pMEK and **I.** CC3 were quantified for cohort comparison.

DTA was evaluated at varied timepoints after parent erlotinib therapy using the iPAI probe pairs at the selected optimal dose (2.5 mg/kg) and imaging timepoint following iPAI probe administration (240 min [4 h]). iPAI probes were administered 6, 12 and 24 h after a therapeutic dose of the parent erlotinib (50 mg/kg) was administered to HCC827 xenograft bearing mice. The goal of these studies was to evaluate the relationship between DTA and biomarkers of the EGFR signaling cascade (i.e., EGFR, pEGFR, Akt, pAkt, MEK, pMEK) as well as viability (i.e., Ki-67 and cleaved caspase 3 [CC3]). Biomarker expression z-scores for all cells in each tissue sample were calculated and single-cell data displayed as violin plots for each timepoint after parent drug + iPAI probe administration to visualize expression level and distribution for each cohort (Fig. 5). The erlotinib target, EGFR, and its phosphorylation measured by pEGFR, showed minimal change in expression across all treatment cohorts (Fig. 5A & 5B). In contrast to pEGFR, DTA displayed a decrease in its median (white dot) at the 12 h timepoint with a shift in the distribution to a greater proportion of cells having below median value DTA in comparison to the distributions in the No Treatment (No Tx) and 6 h timepoints (Fig. 5C). The 24 h cohort exhibited DTA similar to that of the No Tx and 6 h cohorts. Additionally, the EGFR signaling cascade markers Akt and pAkt showed minimal expression change across all timepoints with similar median values (white dot) and expression distribution represented by the shape of the violin plot (Fig. 5D & 5E). Ki67, a marker of proliferation, remained similar in expression across all treatment timepoint groups as well (Fig. 5F). In contrast to Akt and pAkt, EGFR signaling cascade markers MEK and pMEK showed more variable expression across timepoints largely in the 6 and 24 h treatment groups, which showed lower median value expression while expression distribution remained similar across treatment timepoints (Fig. 5G & 5H). Notably CC3, a marker of apoptosis, showed a marked increase in expression in the 12 and 24 h treatment cohorts, where both cohorts displayed similar expression levels and distribution to one another in contrast to the expression level and distribution in the earlier timepoint cohorts (Fig. 5I).

**Figure 6:**
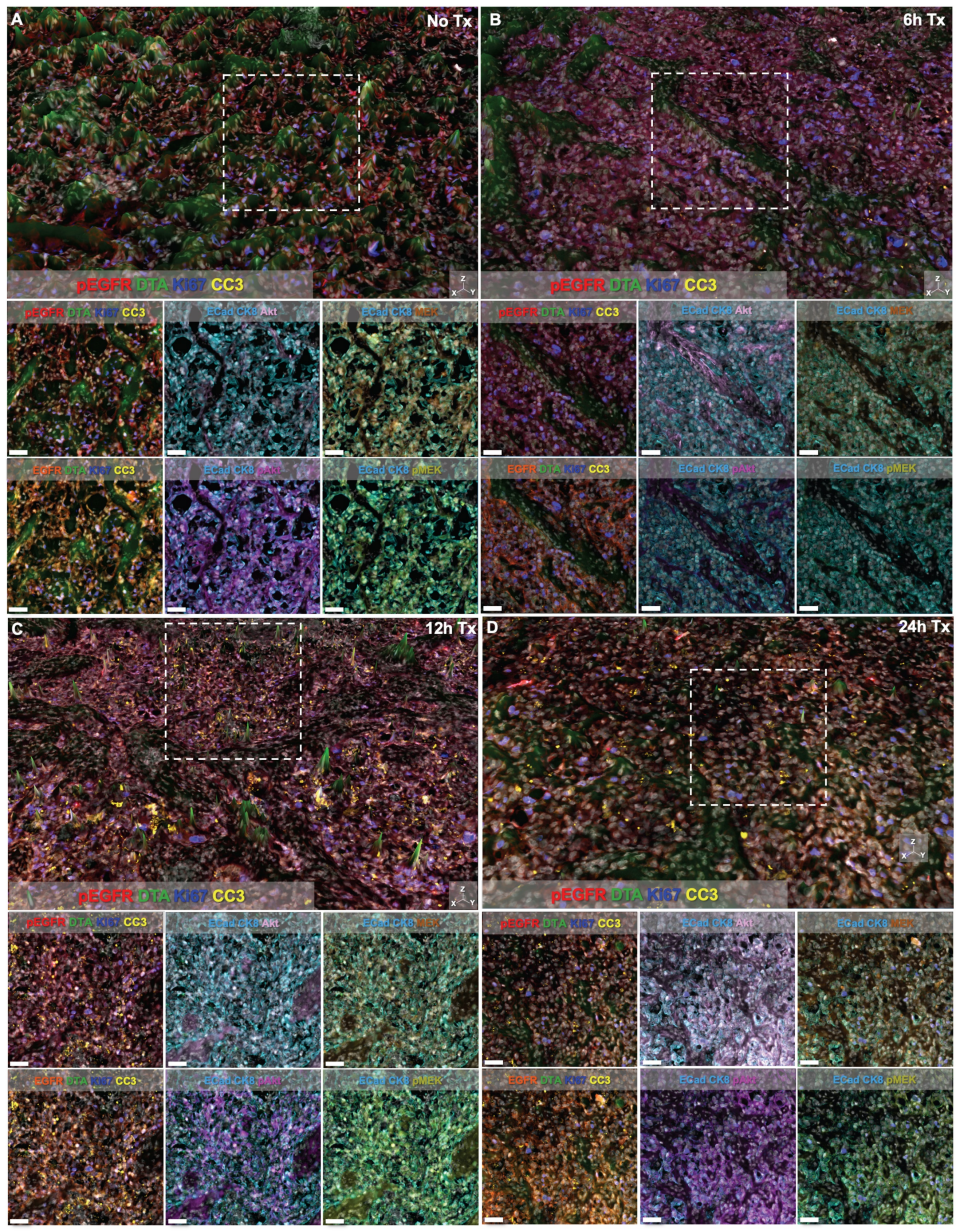
*TRIPODD imaging of control and erlotinib treated tissues.* Representative regions of interest (ROI) from whole tissue images of HCC827 xenografts imaged with multiplexed cyCIF and iPAI are displayed based on treatment cohorts of **A.** No treatment (No Tx), **B.** 6h Tx, **C.** 12h Tx and **D.** 24h Tx. The top panel of each cohort's image set is presented with DTA as a 3D height map where DTA can be visualized both by its green pseudo color and the height of the pixels. DTA height map scaling is applied equivalently across all treatment cohorts. The images below the 3D height map panel show 2D representations of the ROI outlined by the white dashed box in the 3D image where all measured biomarkers are visualized. Markers displayed in each image are set to equivalent contrast settings across treatment groups. Nuclear DAPI signal is displayed in white in all images. Scale bar = 50 μm.

Signal intensity of all markers was also visually assessed in representative regions of interest (ROIs) from each treatment time cohort (Fig. 6). Height maps of DTA signal permitted three-dimensional (3D) visualization of DTA intensity, where the height per pixel represented the quantified level of DTA per pixel. Height maps were scaled equivalently across cohorts for qualitative comparison (Fig. 6, top images per panel). Additionally, the contrast level of each immunostained biomarker and DTA was set equivalently across treatment time cohorts enabling qualitative assessment of signal intensity in both the 3D height map and the two-dimensional (2D) representative ROI images (Fig. 6, white box in top images depicts ROI for 2D images in bottom rows). Comparison of DTA height maps across cohorts showed that DTA levels were similar in the No Tx, 6h Tx and 24h Tx cohorts (Fig. 6A, 6B and 6D), where there were consistently elevated DTA levels in the height map across the representative ROI. In contrast, the 12h Tx cohort displayed DTA expression topography that was flatter than all other cohorts, indicative of lower DTA levels (Fig. 6C). Assessment of the signal patterns of DTA, visualized by comparing pseudocolored intensity, in the 3D height maps also showed the lowest DTA signal level in the 12h Tx timepoint. Ki67 and pEGFR expression levels appeared stable across all cohorts in the 3D height maps (Fig. 6A-D), while CC3 was elevated in 12 and 24h Tx 3D images (Fig. 6C & 6D). In the 2D ROIs where DTA was not displayed, E-Cad and CK8 were pseudocolored the same color to provide a tumor area map for each image. Comparison of the remaining immunostained biomarkers revealed that no major change in expression of the EGFR signaling cascade proteins (i.e., EGFR, pEGFR, Akt, pAkt, MEK and pMEK) was observed in these representative ROIs in any treatment time cohort (Fig. 6). Importantly, the qualitative comparison of signal intensity for all markers is in alignment with the quantified results (Fig. 5 & 6).

## Discussion

Targeted therapeutics (e.g., erlotinib) that aim to inhibit molecular dependencies present in tumors often outperform previous standard of care therapies (e.g., chemo- and radiation-therapy), improving overall patient quality of life and survival outcomes. However, therapeutic response to targeted therapies is rarely durable as tumors evolve to escape therapeutic inhibition through various routes, resulting in the outgrowth of resistant subpopulations of cancer cells. Crucially, current tumor analysis methodologies are unable to unravel the multifaceted routes of therapeutic failure, particularly insufficient drug delivery and cell signaling pathway reprogramming as mechanisms of therapeutic resistance. Our previously reported novel fluorescence imaging platform, TRIPODD, is capable of generating a mechanistic understanding of therapeutic response and resistance that correlates DTA and proteomic therapeutic response at the single cell level to inform on therapeutic strategy design. Optimization of iPAI and extension of the TRIPODD platform to analyze erlotinib therapeutic response at varied times after parent drug treatment was preformed herein as a proof-of-concept demonstration of the TRIPODD platform to quantify therapeutic response (Fig. 1). The validated TRIPODD methodology could be used in future studies to unravel the mechanism(s) of treatment response and resistance, with a focus on developing combination therapy that target resistant cell populations to improve cancer treatment durability.

The optimal timepoint to resect tissues after systemic iPAI probe administration was evaluated through a pharmacokinetic profile study. Distribution of the iPAI probes and calculated DTA at varied timepoints after injection (15, 30, 60, 120, 240, or 480 min) were quantified to determine differences in signal intensity over time and maximize dynamic range of the DTA metric (Fig. 2). Variation was observed in both DTA and fluorescence intensity between and within timepoints, but only tumors resected after 60 and 240 min had both targeted and untargeted median fluorescence intensities above the quantified level of tissue autofluorescence in the vehicle injected control group (Fig. 2B & 2C). Notably, the only timepoint with fluorescence intensity significantly greater than autofluorescence in the vehicle injected control group was the 240 min targeted probe group (Fig. 2B). Also, the highest median DTA was calculated at the 240 min timepoint, which led to its selection as the optimal timepoint to resect tissue after iPAI administration (Fig. 2D). Furthermore, this selected timepoint was also in alignment with previously reported pharmacokinetic profile for erlotinib where 240 min (4 h) was the time required for the drug to reach C_max_ in plasma 82.

Xenografts with varied EGFR expression levels (HCC827 > PANC-1 > SW620) that were systemically administered varied doses of iPAI probes displayed a dose dependent relationship between median targeted and untargeted probe fluorescence intensity (Fig. 3D & 3E). Notably, the lowest tested dose of 1.25 mg/kg resulted in tissues with fluorescence intensity near that of tissue autofluorescence as quantified from the vehicle injected control group. Furthermore, the low EGFR expressing SW620 tissues showed high targeted and untargeted fluorescence intensity signals at the highest dose (5 mg/kg), suggesting that quantitative imaging may not be possible at this dose (Fig. 3D-F). Importantly, the iPAI based correction for non-specific uptake was observed in the 2.5 mg/kg group, where the highest EGFR-expressing tissue, HCC827 (Fig. 3G), yielded DTA values greater than that of the other lower EGFR expressing xenograft tissues, resulting in quantitative imaging of EGFR at this dose (Fig. 3F). To confirm that 2.5 mg/kg was the optimal iPAI dose, a study to correlate EGFR and pEGFR expression to iPAI signal in all analyzed tissues was performed (Fig. 4). Both EGFR and pEGFR expression were significantly correlated to DTA and targeted signal in the 2.5 mg/kg group, while no correlation was seen for any other dose group, further supporting the selection of this dose as optimal for iPAI probe administration (Fig. 4A, 4B, 4D & 4E). No correlation between EGFR or pEGFR expression was seen for any dose of the untargeted iPAI probe, which was expected due to the designed non-specific accumulation of the untargeted probe (Fig. 4C & 4F). Additionally, the slopes of the majority of the calculated trendlines from the untargeted iPAI probe fluorescence intensity were low, signifying that untargeted probe signal alone had minimal relationship to expression of the targeted protein, EGFR.

Further analysis of only EGFR-positive xenografts (i.e., HCC827 and PANC-1) collected in the pharmacodynamic assessment of iPAI probes allowed for comparison of the fluorescence intensity for each iPAI probe in each dose cohort in comparison to a vehicle injected control group (Fig. 4G-I). The HCC827 and PANC-1 xenografts were analyzed without the including of SW620 xenografts since they had minimal EGFR expression. Given their low protein target expression, inclusion of the SW620 xenografts in this analysis could skew comparison of iPAI probe uptake, particularly for the targeted probe, since there were inherently few EGFR binding sites which may result in low targeted probe fluorescence intensity or fluorescence intensity due to non-specific accumulation of the targeted probe. The resulting analysis of HCC827 and PANC-1 xenografts administered at varied doses of iPAI probes revealed that the 1.25 mg/kg cohort did not have targeted or untargeted fluorescence intensity significantly greater than vehicle autofluorescence (Fig. 4G & 4I) and thus was a suboptimal iPAI probe dose. The targeted probe fluorescence intensity of the 2.5 mg/kg and 5 mg/kg dose cohorts were not significantly different, suggesting the targeted iPAI probe binding sites become saturated near the 2.5 mg/kg dose (Fig. 4G). However, the untargeted probe fluorescence intensity was significantly greater in the 5 mg/kg dose cohort than all other cohorts (Fig. 4H). Therefore, while targeted probe binding sites may be saturated at doses >2.5 mg/kg, continued non-specific accumulation of the untargeted probe may negatively impact the accuracy of the DTA calculation. As a result of continued untargeted probe uptake in the 5 mg/kg cohort, the median DTA of the 2.5 mg/kg was significantly greater than the 5 mg/kg median DTA (Fig. 4I). It should also be noted that the while the calculated DTA values in the 2.5 mg/kg and 5 mg/kg dose cohorts were significantly different, this difference could be corrected by scaling cohorts equivalently, so greater DTA alone could not distinguish the 2.5 mg/kg as the optimal iPAI dose. However, when the DTA was put into the context of the calculated Pearson's correlation coefficients, 2.5 mg/kg was shown to be the optimal iPAI probe dose, where iPAI probe fluorescence intensity was significantly above vehicle autofluorescence, while biologically correlative to targeted probe uptake and DTA calculations. Given these combined factors, 2.5 mg/kg was selected as the optimal dose for iPAI administration to facilitate accurate DTA calculations.

Extension of TRIPODD to assess therapeutic response to erlotinib over a 24 h period following a single therapeutic dose was performed on HCC827 xenografts that were collected 6, 12 or 24 h after erlotinib treatment and compared to control, untreated tissues. TRIPODD analysis of these tissues was enabled by iPAI probe injection coupled with a flexible Ab-oligo cyCIF approach integrating indirect IF, direct IF and the Ab-oligo reagents. While a measurable therapeutic effect of erlotinib within 24 h of treatment has been reported 83, quantification of the immunostaining showed no measurable change in pEGFR or total EGFR, the molecular target of erlotinib, at any treatment timepoint (Fig. 5A & 5B). Analysis of downstream EGFR signaling cascade proteins further supported this finding, where no substantial change in downstream protein expression was observed after erlotinib treatment. For example, MEK and pMEK displayed decreased expression at the 6 and 24 h treatment timepoints, but not 12 h after treatment. Importantly, expression distribution, as visualized using violin plots, remained stable across treatment timepoints and was similar to control tissue expression distribution suggesting there were not unique populations of cells reacting to erlotinib treatment.

While the EGFR signaling cascade proteins appeared largely unaffected by erlotinib treatment, CC3, an apoptosis marker, showed a clear increase beginning at the 12 h timepoint and remained elevated at the 24 h timepoint (Fig 5I). This observation was also supported in representative images where CC3 signal was more abundant 12 and 24 h after treatment when qualitatively compared to control tissues and tissues treated for 6 h in representative ROIs (Fig. 6). CC3, when expressed, represents proteolysis during apoptosis and its expression signifies a binary switch of a viable cell to an apoptotic cell in contrast to the gradient of expressions more typical for EGFR cell signaling proteins. Thus, one explanation for this measurable increase in CC3 signal was the high signal intensity and punctate staining pattern that is typical for CC3 as measured by IF. Therefore, by scaling markers by z-score in this analysis, positive CC3 cells demonstrated a marked increase above median control tissue CC3 signal, which were largely negative for CC3. Additionally, CC3 was measured with the Ab-oligo amplification strategy, further increasing sensitivity to its expression 74.

Notably, while therapeutic response measured by changes in pEGFR, EGFR and downstream protein expression did not reveal signatures of erlotinib treatment in any treatment cohort, DTA decreased at the 12 h timepoint and then returned to baseline control tissue DTA level at 24 h (Fig. 5C). This suggests that erlotinib was bound to its EGFR target at 12 h, where a decrease in DTA signified more EGFR binding sites were occupied by the previously administered parent erlotinib. At the 24 h timepoint, the parent erlotinib had cleared from the tissues, resulting in a return of DTA to baseline levels. This suggests the DTA metric was more sensitive to the presence of bound parent erlotinib drug than EGFR expression or its signaling proteins' expression levels. This DTA change was also visualized in representative ROIs from each treatment timepoint cohort, where comparison of DTA can be qualitatively performed based on 3D height maps of DTA expression or 2D images where height map scaling and contrast levels for DTA were set equivalently (Fig. 6). The 3D height map of DTA from the No Tx sample demonstrated baseline DTA topography, where there were many areas of elevated DTA present in the ROI (Fig. 6A). The DTA signal pattern (green) and pEGFR signal intensity (red) also provided a baseline measure of both markers in these control tissues where erlotinib was not present. Across all other timepoints where erlotinib treatment occurred, pEGFR signal intensity remained similar while DTA showed a marked decrease in the 12h Tx ROI, but then a return to baseline levels in the 24h Tx ROI (Fig. 6B-D). Additionally, assessment of DTA topography, demonstrated that DTA was largely low across the ROI in the 12h Tx ROI with only small visible peaks (Fig. 6C). In comparison to the DTA topography of the control tissue as well as the 6 and 24h Tx ROI, the 12 h Tx DTA topography was notably flatter, indicative of widespread bound erlotinib in the tumor tissue. This observation was in alignment with the known pharmacokinetic profile of 50 mg/kg parent erlotinib treatment after a single-dose, where the plasma half-life is 25.5 h 84. Further at 150 mg/kg parent erlotinib dosing, the pharmacokinetic profile of erlotinib does not reach steady state until 7-8 days after daily administration of the drug, providing the rationale for the daily dosing regimen of erlotinib to generate sustained EGFR inhibition 82, 85. This further supports the observation that DTA decreased 12 h after a single dose of erlotinib, but then 24 h after treatment a substantial proportion of erlotinib was cleared from plasma permitting DTA increase to close to baseline levels. Thus, the treatment study described herein provides evidence that DTA can serve as a spatially resolved, single-cell metric for quantification of dosing regimens in the target tissue.

Overall, the antibody-based approaches for biomarker labeling (i.e., flexible Ab-oligo cyCIF) were not of sufficient sensitivity to detect any proteomic changes in the EGFR cell signaling cascade after a single-dose of erlotinib. In contrast, the DTA metric had the sensitivity to detect the presence of bound erlotinib. Notably, a variety of immunostaining reagent types were utilized including indirect IF and Ab-oligo reagents with signal amplification that, in theory, provide greater sensitivity to protein expression perturbations due to the greater signal dynamic range. Therefore, in this study, DTA was validated as more sensitive than IF approaches for measuring cell signaling inhibition after a single-dose of parent erlotinib therapy. Subsequent treatment studies involving tumor bearing animals receiving daily treatment with erlotinib will further explore the relationship between DTA, protein expression and therapeutic response.

Limitations of our study include that only a specific therapy, erlotinib was assessed in a single NSCLC cell line with known sensitivity to erlotinib treatment. Thus, the results of our current work demonstrate the potential utility of the TRIPODD platform in this specific proof-of-concept model. While the NSCLC HCC827 xenografts served as a useful model for validation of the TRIPODD methodology, future studies will include more complex model systems (e.g., orthotopic models, genetically engineered mouse models, syngeneic models) to investigate clinically relevant therapeutic strategies. It is important to note that the optimal iPAI probe administration and tissue collection parameters identified in this work are specific to erlotinib. Further extension of the TRIPODD methodology to different therapeutics will require additional iPAI probe synthesis as well as optimization of administration parameters due to drug-to-drug difference in pharmacokinetic and pharmacodynamic profiles. With the use of more complex model systems as well as evaluation of varied therapeutics, Ab-oligo cyCIF antibody panels will also require incorporation of biomarkers to characterize immune and stromal cells to assess the role the tumor microenvironment plays in drug distribution and binding to its molecular target. In summary, optimal iPAI probe dose administration and tissue resection time were identified herein for erlotinib therapy in NSCLC tumor models. Application of these optimal parameters for the TRIPODD platform will enable future analysis of erlotinib response and resistance to molecularly targeted therapy. In future work, the TRIPODD methodology can be expanded to evaluate other therapeutics and varied cancer types to unravel the nuances between drug dosing strategies, drug delivery to the tumor target and therapeutic response.

## Figures and Tables

**Table 1:** Validated antibody panel for multiplex cyCIF imaging.

Biomarker	Protein	Imaging Strategy
**Tumor Area**	CK8 E-Cad	Ab-oligo 2xFL IS Ab-oligo 2xFL IS
**Tumor Viability**	Ki67 CC3	Ab-oligo 2xFL IS Ab-oligo Amp
**EGRF Signaling Pathway**	EGFR pEGFR MEK 1/2 pMEK 1/2 AKT pAKT	Ab-oligo 2xFL IS Indirect IF Direct IF Ab-oligo Amp Ab-oligo Amp Ab-oligo Amp

*Ab-oligo 2xFL IS = oligonucleotide conjugated antibodies visualized using hybridization to a complementary imaging strand (IS) with a fluorophore on each end (2xFL). **Ab-oligo Amp = oligonucleotide conjugated antibodies visualized using hybridization to a complementary amplification (Amp) strand that has multiple repeating locations for a fluorophore labeled imaging strand (IS) to hybridize.

## References

[1] Bhullar KS, Lagaron NO, McGowan EM, Parmar I, Jha A, Hubbard BP, Rupasinghe HPV (2018). Kinase-targeted cancer therapies: progress, challenges and future directions. Mol Cancer.

[2] Recondo G, Facchinetti F, Olaussen KA, Besse B, Friboulet L (2018). Making the first move in EGFR-driven or ALK-driven NSCLC: first-generation or next-generation TKI?. Nat Rev Clin Oncol.

[3] Maemondo M, Inoue A, Kobayashi K, Sugawara S, Oizumi S, Isobe H (2010). Gefitinib or chemotherapy for non-small-cell lung cancer with mutated EGFR. N Engl J Med.

[4] Zhou C, Wu YL, Chen G, Feng J, Liu XQ, Wang C (2011). Erlotinib versus chemotherapy as first-line treatment for patients with advanced EGFR mutation-positive non-small-cell lung cancer (OPTIMAL, CTONG-0802): a multicentre, open-label, randomised, phase 3 study. Lancet Oncol.

[5] Sequist LV, Yang JC, Yamamoto N, O'Byrne K, Hirsh V, Mok T (2013). Phase III study of afatinib or cisplatin plus pemetrexed in patients with metastatic lung adenocarcinoma with EGFR mutations. J Clin Oncol.

[6] Masters GA, Temin S, Azzoli CG, Giaccone G, Baker S Jr, Brahmer JR (2015). Systemic Therapy for Stage IV Non-Small-Cell Lung Cancer: American Society of Clinical Oncology Clinical Practice Guideline Update. J Clin Oncol.

[7] Lim SM, Syn NL, Cho BC, Soo RA (2018). Acquired resistance to EGFR targeted therapy in non-small cell lung cancer: Mechanisms and therapeutic strategies. Cancer Treat Rev.

[8] Cross DA, Ashton SE, Ghiorghiu S, Eberlein C, Nebhan CA, Spitzler PJ (2014). AZD9291, an irreversible EGFR TKI, overcomes T790M-mediated resistance to EGFR inhibitors in lung cancer. Cancer Discov.

[9] Soria JC, Ohe Y, Vansteenkiste J, Reungwetwattana T, Chewaskulyong B, Lee KH (2018). Osimertinib in Untreated EGFR-Mutated Advanced Non-Small-Cell Lung Cancer. N Engl J Med.

[10] Facchinetti F, Proto C, Minari R, Garassino M, Tiseo M (2018). Mechanisms of Resistance to Target Therapies in Non-small Cell Lung Cancer. Handb Exp Pharmacol.

[11] Vyse S, Howitt A, Huang PH (2017). Exploiting Synthetic Lethality and Network Biology to Overcome EGFR Inhibitor Resistance in Lung Cancer. J Mol Biol.

[12] Lovly CM, Shaw AT (2014). Molecular pathways: resistance to kinase inhibitors and implications for therapeutic strategies. Clin Cancer Res.

[13] Simon GM, Niphakis MJ, Cravatt BF (2013). Determining target engagement in living systems. Nat Chem Biol.

[14] Stefaniak J, Huber KVM (2020). Importance of Quantifying Drug-Target Engagement in Cells. ACS Medicinal Chemistry Letters.

[15] Jain RK (2005). Normalization of Tumor Vasculature: An Emerging Concept in Antiangiogenic Therapy. Science.

[16] Jain RK (2013). Normalizing tumor microenvironment to treat cancer: bench to bedside to biomarkers. J Clin Oncol.

[17] Rutkowska A, Thomson DW, Vappiani J, Werner T, Mueller KM, Dittus L (2016). A Modular Probe Strategy for Drug Localization, Target Identification and Target Occupancy Measurement on Single Cell Level. ACS Chem Biol.

[18] Arrowsmith J, Miller P (2013). Trial watch: phase II and phase III attrition rates 2011-2012. Nat Rev Drug Discov.

[19] Allison M (2012). Reinventing clinical trials. Nat Biotechnol.

[20] Dewhirst MW, Secomb TW (2017). Transport of drugs from blood vessels to tumour tissue. Nat Rev Cancer.

[21] Budayeva HG, Kirkpatrick DS (2020). Monitoring protein communities and their responses to therapeutics. Nat Rev Drug Discov.

[22] Jackson HW, Fischer JR, Zanotelli VRT, Ali HR, Mechera R, Soysal SD (2020). The single-cell pathology landscape of breast cancer. Nature.

[23] Keren L, Bosse M, Thompson S, Risom T, Vijayaragavan K, McCaffrey E (2019). MIBI-TOF: A multiplexed imaging platform relates cellular phenotypes and tissue structure. Sci Adv.

[24] Levine JH, Simonds EF, Bendall SC, Davis KL, Amir el AD, Tadmor MD (2015). Data-Driven Phenotypic Dissection of AML Reveals Progenitor-like Cells that Correlate with Prognosis. Cell.

[25] Wagner J, Rapsomaniki MA, Chevrier S, Anzeneder T, Langwieder C, Dykgers A (2019). A Single-Cell Atlas of the Tumor and Immune Ecosystem of Human Breast Cancer. Cell.

[26] Miller MA, Zheng YR, Gadde S, Pfirschke C, Zope H, Engblom C (2015). Tumour-associated macrophages act as a slow-release reservoir of nano-therapeutic Pt(IV) pro-drug. Nat Commun.

[27] Gao M, Nettles RE, Belema M, Snyder LB, Nguyen VN, Fridell RA (2010). Chemical genetics strategy identifies an HCV NS5A inhibitor with a potent clinical effect. Nature.

[28] Honigberg LA, Smith AM, Sirisawad M, Verner E, Loury D, Chang B (2010). The Bruton tyrosine kinase inhibitor PCI-32765 blocks B-cell activation and is efficacious in models of autoimmune disease and B-cell malignancy. Proc Natl Acad Sci U S A.

[29] Cohen MS, Hadjivassiliou H, Taunton J (2007). A clickable inhibitor reveals context-dependent autoactivation of p90 RSK. Nat Chem Biol.

[30] Dubach JM, Kim E, Yang K, Cuccarese M, Giedt RJ, Meimetis LG (2017). Quantitating drug-target engagement in single cells in vitro and in vivo. Nat Chem Biol.

[31] Stadler C, Rexhepaj E, Singan VR, Murphy RF, Pepperkok R, Uhlen M (2013). Immunofluorescence and fluorescent-protein tagging show high correlation for protein localization in mammalian cells. Nat Methods.

[32] Fischman AJ, Alpert NM, Rubin RH (2002). Pharmacokinetic imaging: a noninvasive method for determining drug distribution and action. Clin Pharmacokinet.

[33] Lomenick B, Hao R, Jonai N, Chin RM, Aghajan M, Warburton S (2009). Target identification using drug affinity responsive target stability (DARTS). Proc Natl Acad Sci U S A.

[34] Matthews PM, Rabiner EA, Passchier J, Gunn RN (2012). Positron emission tomography molecular imaging for drug development. Br J Clin Pharmacol.

[35] Martinez Molina D, Jafari R, Ignatushchenko M, Seki T, Larsson EA, Dan C (2013). Monitoring drug target engagement in cells and tissues using the cellular thermal shift assay. Science.

[36] Munteanu B, Meyer B, von Reitzenstein C, Burgermeister E, Bog S, Pahl A (2014). Label-free in situ monitoring of histone deacetylase drug target engagement by matrix-assisted laser desorption ionization-mass spectrometry biotyping and imaging. Anal Chem.

[37] Grimwood S, Hartig PR (2009). Target site occupancy: emerging generalizations from clinical and preclinical studies. Pharmacol Ther.

[38] Schurmann M, Janning P, Ziegler S, Waldmann H (2016). Small-Molecule Target Engagement in Cells. Cell Chem Biol.

[39] Tichauer KM, Wang Y, Pogue BW, Liu JT (2015). Quantitative in vivo cell-surface receptor imaging in oncology: kinetic modeling and paired-agent principles from nuclear medicine and optical imaging. Phys Med Biol.

[40] Pressman D, Day ED, Blau M (1957). The use of paired labeling in the determination of tumor-localizing antibodies. Cancer Res.

[41] Baeten J, Haller J, Shih H, Ntziachristos V (2009). In vivo investigation of breast cancer progression by use of an internal control. Neoplasia.

[42] Liu JT, Helms MW, Mandella MJ, Crawford JM, Kino GS, Contag CH (2009). Quantifying cell-surface biomarker expression in thick tissues with ratiometric three-dimensional microscopy. Biophys J.

[43] Pogue BW, Samkoe KS, Hextrum S, O'Hara JA, Jermyn M, Srinivasan S, Hasan T (2010). Imaging targeted-agent binding in vivo with two probes. J Biomed Opt.

[44] Davis SC, Gibbs SL, Gunn JR, Pogue BW (2013). Topical dual-stain difference imaging for rapid intra-operative tumor identification in fresh specimens. Opt Lett.

[45] Tichauer KM, Deharvengt SJ, Samkoe KS, Gunn JR, Bosenberg MW, Turk MJ (2014). Tumor endothelial marker imaging in melanomas using dual-tracer fluorescence molecular imaging. Mol Imaging Biol.

[46] Tichauer KM, Diop M, Elliott JT, Samkoe KS, Hasan T, St Lawrence K, Pogue BW (2014). Accounting for pharmacokinetic differences in dual-tracer receptor density imaging. Phys Med Biol.

[47] Tichauer KM, Samkoe KS, Gunn JR, Kanick SC, Hoopes PJ, Barth RJ (2014). Microscopic lymph node tumor burden quantified by macroscopic dual-tracer molecular imaging. Nat Med.

[48] Barth CW, Schaefer JM, Rossi VM, Davis SC, Gibbs SL (2017). Optimizing fresh specimen staining for rapid identification of tumor biomarkers during surgery. Theranostics.

[49] Maniwa Y, Yoshimura M, Obayashi C, Inaba M, Kiyooka K, Kanki M, Okita Y (2001). Association of p53 gene mutation and telomerase activity in resectable non-small cell lung cancer. Chest.

[50] Tichauer KM, Samkoe KS, Sexton KJ, Gunn JR, Hasan T, Pogue BW (2012). Improved tumor contrast achieved by single time point dual-reporter fluorescence imaging. J Biomed Opt.

[51] Tichauer KM, Samkoe KS, Sexton KJ, Hextrum SK, Yang HH, Klubben WS (2012). In vivo quantification of tumor receptor binding potential with dual-reporter molecular imaging. Mol Imaging Biol.

[52] Tichauer KM, Holt RW, El-Ghussein F, Davis SC, Samkoe KS, Gunn JR (2013). Dual-tracer background subtraction approach for fluorescent molecular tomography. J Biomed Opt.

[53] Wang D, Chen Y, Leigh SY, Haeberle H, Contag CH, Liu JT (2012). Microscopic Delineation of Medulloblastoma Margins in a Transgenic Mouse Model Using a Topically Applied VEGFR-1 Probe. Transl Oncol.

[54] Meng B, Folaron MR, Byrd BK, Samkoe KS, Strawbridge RS, Barth C (2020). Topical dual-probe staining using quantum dot-labeled antibodies for identifying tumor biomarkers in fresh specimens. PLOS ONE.

[55] House BJ, Schaefer JM, Barth CW, Davis SC, Gibbs SL (2019). Diagnostic Performance of Receptor-Specific Surgical Specimen Staining Correlate with Receptor Expression Level. Proc SPIE Int Soc Opt Eng.

[56] Folaron MR, Strawbridge RR, Samkoe KS, Gibbs SL, Davis SC (2019). Effect of staining temperature on topical dual stain imaging of tissue specimens for tumor identification. Proc SPIE Int Soc Opt Eng.

[57] Schaefer JM, Barth CW, Davis SC, Gibbs SL (2019). Diagnostic performance of receptor-specific surgical specimen staining correlates with receptor expression level. Journal of biomedical optics.

[58] Wang LG, Montano AR, Combs JR, McMahon NP, Solanki A, Gomes MM (2023). OregonFluor enables quantitative intracellular paired agent imaging to assess drug target availability in live cells and tissues. Nat Chem.

[59] Goltsev Y, Samusik N, Kennedy-Darling J, Bhate S, Hale M, Vazquez G (2018). Deep Profiling of Mouse Splenic Architecture with CODEX Multiplexed Imaging. Cell.

[60] Lin JR, Izar B, Wang S, Yapp C, Mei S, Shah PM (2018). Highly multiplexed immunofluorescence imaging of human tissues and tumors using t-CyCIF and conventional optical microscopes. Elife.

[61] Lin JR, Fallahi-Sichani M, Sorger PK (2015). Highly multiplexed imaging of single cells using a high-throughput cyclic immunofluorescence method. Nat Commun.

[62] Levenson RM, Borowsky AD, Angelo M (2015). Immunohistochemistry and mass spectrometry for highly multiplexed cellular molecular imaging. Lab Invest.

[63] Angelo M, Bendall SC, Finck R, Hale MB, Hitzman C, Borowsky AD (2014). Multiplexed ion beam imaging of human breast tumors. Nat Med.

[64] Giesen C, Wang HA, Schapiro D, Zivanovic N, Jacobs A, Hattendorf B (2014). Highly multiplexed imaging of tumor tissues with subcellular resolution by mass cytometry. Nat Methods.

[65] Gerdes MJ, Sevinsky CJ, Sood A, Adak S, Bello MO, Bordwell A (2013). Highly multiplexed single-cell analysis of formalin-fixed, paraffin-embedded cancer tissue. Proc Natl Acad Sci U S A.

[66] Tsujikawa T, Kumar S, Borkar RN, Azimi V, Thibault G, Chang YH (2017). Quantitative Multiplex Immunohistochemistry Reveals Myeloid-Inflamed Tumor-Immune Complexity Associated with Poor Prognosis. Cell Rep.

[67] Feng Z, Jensen SM, Messenheimer DJ, Farhad M, Neuberger M, Bifulco CB, Fox BA (2016). Multispectral Imaging of T and B Cells in Murine Spleen and Tumor. J Immunol.

[68] Lin JR, Fallahi-Sichani M, Chen JY, Sorger PK (2016). Cyclic Immunofluorescence (CycIF), A Highly Multiplexed Method for Single-cell Imaging. Curr Protoc Chem Biol.

[69] Remark R, Merghoub T, Grabe N, Litjens G, Damotte D, Wolchok JD (2016). In-depth tissue profiling using multiplexed immunohistochemical consecutive staining on single slide. Sci Immunol.

[70] Zrazhevskiy P, Gao X (2013). Quantum dot imaging platform for single-cell molecular profiling. Nat Commun.

[71] Glass G, Papin JA, Mandell JW (2009). SIMPLE: a sequential immunoperoxidase labeling and erasing method. The journal of histochemistry and cytochemistry: official journal of the Histochemistry Society.

[72] McMahon NP, Jones JA, Kwon S, Chin K, Nederlof MA, Gray JW, Gibbs SL (2020). Oligonucleotide conjugated antibodies permit highly multiplexed immunofluorescence for future use in clinical histopathology. Journal of Biomedical Optics.

[73] Jones JA, McMahon NP, Zheng T, Eng J, Chin K, Kwon S (2021). Oligonucleotide conjugated antibody strategies for cyclic immunostaining. Sci Rep.

[74] McMahon NP, Jones JA, Anderson AN, Dietz MS, Wong MH, Gibbs SL (2023). Flexible Cyclic Immunofluorescence (cyCIF) Using Oligonucleotide Barcoded Antibodies. Cancers (Basel).

[75] McMahon NP, Solanki A, Wang LG, Montano AR, Jones JA, Samkoe KS (2021). TRIPODD: a Novel Fluorescence Imaging Platform for In Situ Quantification of Drug Distribution and Therapeutic Response. Mol Imaging Biol.

[76] Johnson LN (2009). Protein kinase inhibitors: contributions from structure to clinical compounds. Q Rev Biophys.

[77] Solanki A, Wang L, Korber J, McMahon N, Tichauer K, Samkoe KS, Gibbs SL (2020). Intracellular paired agent imaging enables improved evaluation of tyrosine kinase inhibitor target engagement. Proc SPIE Int Soc Opt Eng.

[78] Higgins B, Kolinsky K, Smith M, Beck G, Rashed M, Adames V (2004). Antitumor activity of erlotinib (OSI-774, Tarceva) alone or in combination in human non-small cell lung cancer tumor xenograft models. Anticancer Drugs.

[79] Abraham J, Nelon LD, Kubicek CB, Kilcoyne A, Hampton ST, Zarzabal LA (2011). Preclinical testing of erlotinib in a transgenic alveolar rhabdomyosarcoma mouse model. Sarcoma.

[80] Chang YH, Tsujikawa T, Margolin A, Coussens LM, Gray JW (2017). Multiplexed immunohistochemistry image analysis using sparse coding. 2017 39th Annual International Conference of the IEEE Engineering in Medicine and Biology Society (EMBC).

[81] Labrie M, Li A, Creason A, Betts C, Keck J, Johnson B (2021). Multiomics analysis of serial PARP inhibitor treated metastatic TNBC inform on rational combination therapies. NPJ Precis Oncol.

[82] Kucharczuk CR, Ganetsky A, Vozniak JM (2018). Drug-Drug Interactions, Safety, and Pharmacokinetics of EGFR Tyrosine Kinase Inhibitors for the Treatment of Non-Small Cell Lung Cancer. J Adv Pract Oncol.

[83] Wu Q, Li MY, Li HQ, Deng CH, Li L, Zhou TY, Lu W (2013). Pharmacokinetic-pharmacodynamic modeling of the anticancer effect of erlotinib in a human non-small cell lung cancer xenograft mouse model. Acta Pharmacol Sin.

[84] Takeda Y, Ishizuka N, Sano K, Hirano S, Suzuki M, Naka G, Sugiyama H (2020). Phase I/II Study of Erlotinib to Determine the Optimal Dose in Patients With Non-Small Cell Lung Cancer Harboring Only EGFR Mutations. Clin Transl Sci.

[85] Lu JF, Eppler SM, Wolf J, Hamilton M, Rakhit A, Bruno R, Lum BL (2006). Clinical pharmacokinetics of erlotinib in patients with solid tumors and exposure-safety relationship in patients with non-small cell lung cancer. Clin Pharmacol Ther.

